# Physical and psychosocial factors associated with sexual satisfaction in long-term cancer survivors 5 and 10 years after diagnosis

**DOI:** 10.1038/s41598-023-28496-1

**Published:** 2023-02-03

**Authors:** Svenja Heyne, Sabine Taubenheim, Andreas Dietz, Florian Lordick, Heide Götze, Anja Mehnert-Theuerkauf

**Affiliations:** 1 Department of Medical Psychology and Medical Sociology, University Medical Center Leipzig, Philipp-Rosenthal-Str. 55, 04103 Leipzig, Germany; 2 Clinical Cancer Registry Leipzig, University Medical Center Leipzig, Leipzig, Germany; 3 Department of Otolaryngology, Head and Neck Surgery, University Medical Center Leipzig, Leipzig, Germany; 4 Division of Oncology, Department of Medicine, University of Leipzig Medical Center, Leipzig, Germany

**Keywords:** Cancer, Psychology, Health care, Oncology

## Abstract

Our study provides data on sexual satisfaction among long-term cancer survivors 5 and 10 years after diagnosis, and identifies factors detrimental (e.g. psychosocial and physical symptom burden) or beneficial (e.g. social support) to survivors’ sexual satisfaction. We measured sexual satisfaction among cancer survivors recruited via the local clinical cancer registry across a wide range of tumor sites 5 years (cohort 1) and 10 years (cohort 2) after diagnosis. We further assessed chronic comorbidity index (CCI) and symptom scales (EORTC QLQ-C30), depression (PHQ-9) and anxiety (GAD-7), satisfaction with partnership (PFB), quality of life (EORTC QLQ-C30), and social support (OSSS). 924 patients (5‐year cohort = 608/10‐year cohort = 316) participated in the study (53% men, 80% cohabiting, mean age 66 years, range 18–85). We found that nearly half of the respondents perceived their sexual life as less satisfying than before cancer. High sexual satisfaction was associated with a low chronic comorbidities index (*r* = − 0.27, *p* < .001),  less fatigue (*r* = − 0.35, *p*<.001), less nausea/vomiting (*r* = − 0.13, *p*<.001) and less pain (*r* = − 0.23, *p*<.001), *r *; less depression (*r* = − 0.24, *p* < .001), less anxiety(*r* = − 0.23, *p* < .001); a high level of social support (*r* = 0.16, *p* < .001), a high level of satisfaction with their relationship (*r* = 0.24, *p* < .001), and high quality of life (*r* = 0.33, *p* < .001). Sexual satisfaction may be affected by both psychosocial and physical symptom burden, with the latter having a greater impact on sexual satisfaction. It is essential for health care providers that sexual health issues are understood, evaluated, and treated, including those of long-term cancer survivors.

## Introduction

With advances in early detection and treatment of cancer alongside the aging and growth of the population, the number of cancer survivors continues to increase^1^. As research on late and/or long-term effects has shown, cancer survivors often face the consequences of their cancer diagnoses and treatments, which can influence various areas of their lives, even years after treatment is completed^2,3^.

Sexual health is a key component of overall physical and psychosocial well-being^4^ and overall relationship quality^5–7^, yet is often impaired in cancer survivors^8–10^. In contrast to many other side effects of cancer treatment, sexual problems do not usually diminish during the first two years of survival, but can remain constant and relatively severe^11^. As research points out, cancer survivors have a greater prevalence and persistence of sexual problems than healthy individuals of the same age^6,12,13^.

Sexual satisfaction is a person's subjective report of positive sexual experiences; a sense of satisfaction with sexual activity, sexual enjoyment, sexual functioning and sexual intimacy^8^. An English study compared 193 cancer survivors with 2831 cancer-free controls regarding their satisfaction with their overall sex life and found significant lower sexual satisfaction in both women and men with cancer diagnoses compared to the cancer-free controls^14^.

In portraying sexual health of cancer survivors, previous research has primarily focused on the biomedical perspective of sexual (dys)function and/or has been clinically oriented^7^. However, sexual satisfaction is not necessarily related to specific aspects of sexual function, as satisfaction with sex life and intimacy can exist despite impaired sexual function^15^. Factors that are associated with sexual (dys-)function have been studied extensively, e.g. the type of therapy and other disease-specific factors^16–20^, physiological effects (e.g. physical comorbidities)^21–25^ and long-term psychosocial effects (e.g. anxiety, depression, low quality of life and low social support)^26–30^. With some exceptions^31,32^, less is known about the factors that are associated with sexual satisfaction in long-term cancer survivors with a cancer diagnosis across all entities.

To address this gap in existing research, this study focused on gaining insight into survivors’ sexual satisfaction. In addition to physical factors, we explore psychosocial factors that may contribute to individual satisfaction. Variables such as psychosocial and physical well-being, quality of life, and relationship satisfaction may be more crucial than medical or demographic factors in affecting the risk of sexual dissatisfaction.

Our research questions were as follows:

1. What is the prevalence of sexual satisfaction in long-term cancer survivors (1) 5 and 10 year after diagnosis? (2) living with or without a partner?
2. Is sexual satisfaction (1) associated with physical symptom burden and psychological distress? (2) Are there protective factors or inter- and intrapersonal resources, (e.g. social support, quality of life)?

## Methods

### Study design and sample

In this cross-sectional cohort study, we recruited patients diagnosed with cancer 5 or 10 years before through the Clinical Cancer Registry Leipzig, Germany^33^. We selected the first cohort at 5 years after the primary cancer diagnosis, as the most common definition of long-term survival refers to a timespan of at least five years since diagnosis^34^, and the second cohort 10 years after the primary cancer diagnosis, to capture late- and long-term conditions after the usual follow-up periods.

Patients were eligible for participating in the study if they had a confirmed diagnosis of cancer 5 years (*cohort 1*) or 10 years (*cohort 2*) before, were at least 18 years of age at the time of diagnosis, were up to 85 years of age at the time of assessment, and were fluent in written and spoken German. All participants gave written informed consent in accordance with the Declaration of Helsinki. The study was approved by the Research Ethics Committee of the University of Leipzig (Ref. 070-14-10032014).

### Study recruitment and data collection

Access to patients was provided by the cancer registry at the Leipzig Cancer Center. The clinical cancer registry provided data on sex, age, ICD-10 diagnosis, time of diagnosis, and cancer treatments received. Trained cancer registry staff selected patients who both gave general consent to be contacted for research projects and met our inclusion criteria. Previously deceased patients could be identified by registry staff, if they had died at the Leipzig Cancer Center. On a monthly basis, patients were identified by registry staff using the inclusion criterion “time since diagnosis: 5 or 10 years ago”. Those patients received a study information letter and were asked to participate in the survey by the research team. If there was no response, there were a maximum of two reminders. A postage-paid response card was included. Patients who consented to participate received the questionnaire by mail or could complete it online using the software *Lime Survey.* Eligible patients who refused to participate were asked to provide their reason for non-participation.

### Study measures

#### Sociodemographic and clinical data

Sociodemographic characteristics, i.e., sex, age, marital status, living with a partner, education, household income per month were obtained from patients’ self-reports. Clinical characteristics, i.e. cancer diagnosis, cancer recurrence, metastases, second cancer disease as well as received treatments could be recorded based on patients’ medical charts. Some of these data were also available through the cancer registry and were additionally collated for verification purposes.

#### Sexual satisfaction

Based on clinical experience, a new questionnaire was developed to assess sexual satisfaction in cancer survivors. This questionnaire included 11 items: five to be completed by all respondents, three only by respondents who are currently in a committed partnership and a further three items by respondents who are not currently in a committed partnership. Higher scores were indicating higher sexual satisfaction. The questionnaire is appended in the supplementary material. Due to missing reference data and to aid interpretability, the scales on sexual satisfaction were recoded as shown in Table 1.

**Table 1:** Recoding procedure for the questionnaire on sexual satisfaction.

Response category	Code old	Response category	Code new
Strongly unsatisfied, unsatisfied	1, 2	Unsatisfied	1
Always, almost always	1, 2	Frequent	1
Disagree at all, disagree	1, 2	Disagree	1
Much worse, rather worse	1, 2	Worse	1
Undecided, sometimes	3	Undecided	2
Satisfied, extremely satisfied	4, 5	Satisfied	3
Rare, never	4, 5	Infrequent	3
Agree, fully agree	4, 5	Agree	3
Rather better, better	4, 5	Better	3

#### Physical symptom burden

##### Chronic comorbidity index

For assessing physical comorbidities, we used a modified version of a self-report instrument^33^ developed by Bayliss et al.^35^. The original chronic comorbidity index (CCI) comprises a list of 23 common chronic medical conditions. The specificity of the original scale was reduced by combining similar conditions (e.g., angina/coronary artery disease and congestive heart failure were combined under ‘heart disease’. We modified the instrument to be more cancer-specific: (1) the condition ‘cancer’ was removed as our sample only consists of cancer survivors, (2) two further conditions were added: psychological diseases, because the main study was primarily concerned with psychological long-term consequences in cancer survivors, and polyneuropathy, as this is a common short and long-term consequence of cancer and its treatment.

Respondents reported for each of the 18 conditions whether they had the condition and, if so, whether it interfered with their daily activities on a scale from ‘not at all’ (a weight of 1) to ‘a lot’ (a weight of 5). Weighting each reported condition by the degree of limitation yields a measure of "disease burden" (comorbidity index). The total score ranges between 0 and 90 and represents the sum of conditions weighted by the level of interference assigned to each^36^.

##### Symptom scales and physical functioning

For analyzing physical symptom burden as a facet of health-related quality of life, we used the three symptom scales (fatigue, pain and nausea/vomiting) and the functioning scale of the German version of the European Organization for Research and Treatment of Cancer Quality of life Questionnaire (EORTC QLQ-C30)^37^. All of the scales range from 0 to 100. A high score for a symptom scale represents a high level of symptomatology/problems and a high score on the physical functioning scale represents a high/healthy level of physical functioning^38^.

#### Psychological symptom burden

##### Depression and general anxiety disorder symptomatology

We used the modules for depression (PHQ-9) and general anxiety disorder (GAD-7) from the validated German version of the Patient Health Questionnaire (PHQ)^39^. Respondents rated the frequency of symptoms on a four-point Likert scale ranging from “not at all” to “almost every day”. The sum score of the PHQ-9 ranges from 0 to 27 and for the GAD-7 from 0 to 21 with scores of ≥ 5, ≥ 10 and ≥ 15 indicating mild, moderate and severe symptoms respectively.

##### Emotional and cognitive functioning

We used the emotional and cognitive functioning scale of the German version of the European Organization for Research and Treatment of Cancer Quality of life Questionnaire (EORTC QLQ-C30)^37^. All of the scales range from 0 to 100. A high score on each of the functioning scales represents a high/healthy level of emotional or cognitive functioning^38^.

#### Inter- and intrapersonal resources

##### Satisfaction with the relationship

For assessing satisfaction with the relationship or the marriage we used the "happiness item" ("How happy do you consider your partnership to be?") from the Relationship Questionnaire (Partnerschaftsfragebogen—PFB)^40^. Respondents can rate the quality of their relationship from 0 (“very unhappy”) to 5 (“very happy”).

##### Functioning scales and global quality of life

For assessing functioning and general quality of life, we used the two functioning scales (role and social) and the global health status scale of the German version of the EORTC QLQ-C30^37^. All of the scales scores range from 0 to 100. A high score for a functional scale represents a high/healthy level of functioning, a high score for the global health status/quality of life indicates a high quality of life^38^.

##### Social support

For assessing perceived social support, we used the Oslo Social Support Scale (OSSS)^41,42^. Respondents can indicate how many people close to them they have, how much interest they receive from others, and how much practical help they receive. The sum score is ranging from 3 to 14 with a score from 3 to 8, 9 to 11 and 12 to 14 indicating low, medium and high social support respectively^43^.

### Statistical analysis

We applied descriptive analyses for both continuous (frequencies, mean, standard deviation) and categorical variables (frequencies, percentages).

To examine differences between cohorts (5 vs. 10 years time since diagnosis) in demographic and clinical variables, chi-square tests were calculated for all categorical variables and a t-test was calculated for the continuous variable age. Comparisons between cohorts or between participants and non-responders were performed in a one-way analysis of variance (ANOVA) with Bonferroni correction due to multiple comparisons (adjusted α level 0.00625). Linear correlations between two variables were examined with bivariate correlation using Pearson’s r with the following guidelines for interpreting effect sizes: small = 0.10, medium = 0.30 and large = 0.50^44^. For identifying robust and independent associated factors, we performed linear regression models entering the variables identified in former ANOVA and correlation matrices in 5 blocks with sexual satisfaction as the dependent variable. The following blocks were entered: sex (Block 1—sociodemographic data); chemotherapy, radiation, hormone therapy (Block 2—clinical data); chronic comorbidity index, fatigue, pain and nausea/vomiting, physical functioning (Block 3—physical symptom burden); anxiety, depression, emotional functioning and cognitive functioning (Block 4—psychological symptom burden) and partnership satisfaction, social support, social functioning, roles functioning, global quality of life (Block 5—inter- and intrapersonal resources).

Predictors were checked for multicollinearity with bivariate correlation analysis and calculating the variance inflation factor (VIF) resulting in significant high correlations between anxiety and depression (*r* = 0.82, VIF = 3.1) and role function and physical function (*r* = 0.75, VIF = 2.3). We decided to include all predictors in the regression model, since the sample size is large enough and VIF is under a critical threshold of 5^45^. Data analyses were performed with IBM SPSS Statistics 27^46^.

### Ethical approval

All procedures performed were in accordance with the ethical standards of the institutional and/or national research committee and with the 1964 Helsinki declaration and its later amendments or comparable ethical standards. Approval was granted by the Ethics Committee of University of Leipzig (Az. 070-14-10032014).

### Consent to participate

Informed consent was obtained from all individual participants included in the study.

## Results

### Sample

**Figure 1:**
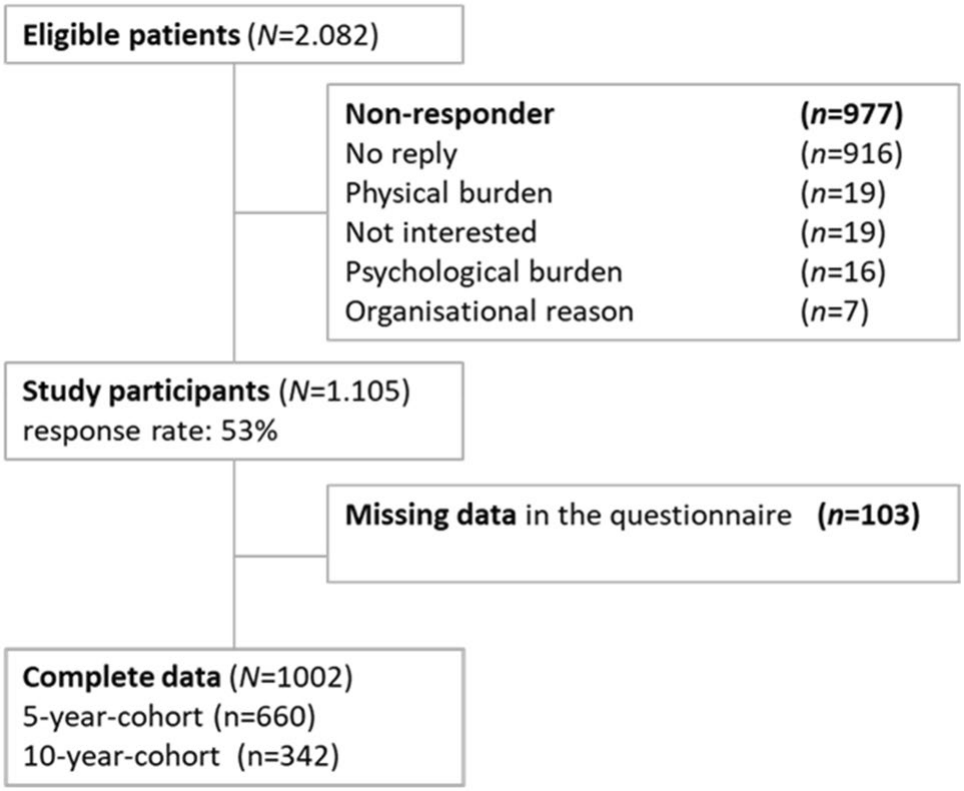
Flowchart of participants.

Patient recruitment was carried out from October 2014 to November 2015. Out of 2082 eligible patients (5‐year cohort *n* = 1396, 10‐year cohort *n* = 686), 1105 (response rate = 53%) participated in the study (Fig. 1).

A total of 1002 patients returned a complete questionnaire: 5-year cohort *n* = 660 (65.9%), 10-year cohort *n* = 342 (34.1%) and were included in the final analysis (postal participation: *n* = 758, online participation: *n* = 244). Patients who completed the questionnaires online were younger (*M* = 60.8 years; *SD* = 12.5; *t*(831) = 7.75, *p* < 0.001) and male (63.6%; *χ*^*2*^(1) = 5.62, *p* = 0.019) compared to those who completed the questionnaire in the paper–pencil version.

#### Non-responder analysis

Study participants were more likely to be male (53% vs. 47%, *p* = 0.013). They differed in cancer type (*p* = 0.001), with a higher percentage of prostate cancer (25% vs. 16.5%) and lower percentage of skin cancer (5.8% vs. 7.8%) and colon cancer (4.7% vs. 6.0%) compared to non-responders. There were no significant age differences between both groups (non-responder *M* = 65.8 years, *p* = 0.054).

Table 2 shows sociodemographic and clinical data for the total sample and separately for the two cohorts (5 and 10 years after diagnosis). The majority of the participants were 50 years and older (93.1%) and lived together with their partner (79.8%). Almost three quarters (71.1%) of respondents had attended school for ten or more years and about half (44.3%) of the respondents had a household income of 1500–2500 €/month. The two most common cancer diagnoses were prostate (25.5%) and breast cancer (21.8%). One in ten had recurrence and metastasis and one in five had another cancer diagnosis.

**Table 2:** Characteristics for the total sample and both cohorts 5 and 10 years after cancer diagnosis.

	Total Sample	5-year-cohort	10-year-cohort	*p*
	*n* (valid %)	*n* (valid %)	*n* (valid %)	
	1002 (100)	660 (65.9)	342 (34.1)	
*Sociodemographic data*				
Sex				.947
Male	530 (52.9)	350 (34.9)	180 (18.0)	
Female	472 (47.1)	310 (30.9)	162 (16.2)	
Age in years, M (SD)	66.7 (10.5)	66.3 (10.5)	67.6 (10.4)	.052
18–49	69 (6.9)	49 (4.9)	20 (2.0)	
50–70	468 (46.7)	325 (32.4)	143 (14.3)	
71–85	465 (46.4)	286 (28.5)	179 (17.9)	
Marital status^a^				.893
Single	64 (6.4)	45 (4.5)	19 (1.9)	
Married	706 (70.7)	460 (46.1)	246 (24.6)	
Divorced	110 (11.0)	75 (7.5)	35 (3.5)	
Widowed	105 (10.5)	68 (6.8)	37 (3.7)	
Living with a partner^b^				.356
Yes	777 (79.8)	504 (51.7)	273 (28.0)	
No	197 (20.2)	135 (13.9)	62 (6.4)	
Highest secondary education^c^				.166
Elementary school (8–9 y)	284 (28.4)	185 (18.5)	99 (9.9)	
Junior high school (10 y)	336 (33.6)	235 (23.5)	101 (10.1)	
High school (12–13 y)	61 (6.1)	44 (4.4)	17 (1.7)	
University	314 (31.4)	191 (19.1)	123 (12.3)	
Other	5 (0.5)	4 (0.4)	1 (0.1)	
Household income in EUR				.002*
< 1500	336 (33.5)	246 (24.6)	90 (9.0)	
1500–2500	444 (44.3)	281 (28.0)	163 (16.3)	
> 2500	222 (22.2)	133 (13.3)	89 (8.9)	
*Clinical data*				
Cancer Diagnosis^d^				< .001*
Prostate	255 (25.5)	175 (17.5)	80 (8.0)	
Breast	218 (21.8)	156 (15.6)	62 (6.2)	
Gynaecological	95 (9.5)	59 (5.9)	36 (3.6)	
Head and neck	78 (7.8)	53 (5.3)	25 (2.5)	
Hematological	75 (7.5)	38 (3.8)	37 (3.7)	
Skin	58 (5.8)	46 (4.6)	12 (1.2)	
Kindney	50 (5.0)	26 (2.6)	24 (2.4)	
Colon	47 (4.7)	26 (2.6)	21 (2.1)	
Other	125 (12.5)	81 (8.1)	44 (4.4)	
Cancer-related data				
Cancer recurrence^e^	106 (11.1)	63 (6.6)	43 (4.5)	.193
Metastases^f^	104 (10.9)	75 (7.9)	29 (3.0)	.156
Second cancer disease^g^	192 (19.6)	123 (12.6)	69 (7.0)	.611
Received Treatments				
Surgery^h^	877 (91.9)	586 (61.4)	291 (30.5)	.211
Chemotherapy^i^	363 (49.1)	241 (32.6)	122 (16.5)	.977
Radiotherapy^j^	579 (69.0)	384 (45.8)	195 (23.2)	.693
Hormone Therapy^k^	191 (28.5)	138 (20.6)	53 (7.9)	.143

n = sub-sample size, p = level of statistical significance between 5 and 10-year-cohort, ^a^n/a = 4, ^b^n/a = 28, ^c^n/a = 2, ^d^n/a = 1, ^e^n/a = 51, ^f^n/a = 49, ^g^n/a = 22, ^h^n/a = 48, ^i^n/a = 263, ^j^n/a = 163, ^k^n/a = 331.

*Significant on a level of p < .05.

The two cohorts differed significantly in terms of the characteristics of household income and cancer diagnosis. Participants diagnosed ten years before were on average older (*t*(935) = -2.67, *p* = 0.008, *d* = − 0.18), had a higher household income (*χ*^*2*^(2) = 13.83, *p* = 0.001, *V* = 0. 124), were more likely to have hematologic tumors and less likely to have breast cancer (*χ*^*2*^(8) = 24.57, *p* = 0.002, *V* = 0.162), and were less likely to have been treated with hormone therapy (*χ*^*2*^(1) = 5.12, *p* = 0.024, *V* = 0.07) than participants diagnosed five years before.

### Sexual satisfaction

Respondents were mostly satisfied with their physical attractiveness (48.6%). Compared to the time before cancer diagnosis, respondents indicated that they were less satisfied (48.9%) with sexuality at the time of the survey, with 47.5% noting no change for better or worse. For communication about sexuality, 33.6% noted a change for the worse, whereas the majority (58.5%) felt that communication remained the same. The majority of respondents had neither discussed problems and concerns regarding their sexuality with a doctor or psychologist (82.5%) nor felt the need to discuss them (83.1%). Of the respondents living with a partner, 43.8% were satisfied with the sexual relationship with their partner and 28.8% unsatisfied. 27.4% were undecided. More than half of the respondents reported no interference with sexual pleasure: neither by physical symptoms, e.g. pain (51.2%), nor by mental distress, e.g. sadness (56.7%). Of the respondents living without a partner, slightly more were satisfied with their sex life (41.3%) than unsatisfied (35.1%) and 23.6% were undecided. Respondents living without a partner were in roughly equal parts satisfied (42.4%) and dissatisfied (42.0%), respectively, with their life without a partner, and 66.9% of respondents did not wish for a new partner.

The two cohort did not differ significantly in their sexual satisfaction (5 years after diagnosis: *M* = 2.01, *SD* = 0.556; 10 years after diagnosis: *M* = 2.06, *SD* = 0.552, *p *= 0.170). Table 3 shows the means, standard deviations and significance levels of all item responses for the two cohorts (5 vs. 10 years) on sexual satisfaction.

**Table 3:** Means, standard deviations and significance levels of item response.

Cohorts: time since diagnosis	Items on sexual satisfaction			
	*N*	*M*	*SD*	*p*
1 Satisfaction with physical attractiveness				
5 years	580	3.15	1.04	.046*
10 years	302	3.30	0.98	
2^a^ Satisfaction with sexual relationship				
5 years	467	3.08	1.13	.735
10 years	252	3.11	1.15	
3^a^ Sexual impairment (due to physical symptoms)				
5 years	168	3.12	1.42	.052
10 years	103	2.78	1.38	
4^a^ Sexual impairment (due to mental distress)				
5 years	155	2.99	1.66	.466
10 years	89	2.83	1.50	
5^b^ Satisfaction with sex life				
5 years	158	2.04	1.29	.616
10 years	93	1.96	1.14	
6^b^ Satisfaction with life				
5 years	539	2.26	0.95	.533
10 years	277	2.30	0.94	
7^b^ Wish for new partnership				
5 years	531	2.57	0.91	.509
10 years	267	2.62	0.94	
8 Change in sexual satisfaction				
5 years	442	3.32	1.47	.322
10 years	232	3.44	1.40	
9 Change in communication with sexuality				
5 years	433	3.53	1.36	.420
10 years	230	3.62	1.29	
10 Problems discussed				
5 years	581	–	–	.219
10 years	289	–	–	
11 Need to discuss problems				
5 years	572	–	–	.847
10 years	285	–	–	

^a^Items to be answered when living with a partner.

^b^Items to be answered when living without a partner, *significant on a level of p < .05.

Value range items 1–9 is [1,5] with higher scores indicating higher satisfaction and freedom from impairment, respectively.

Items 1–9 with t-test.

Items 10–11 with χ^2^-test.

### Associations with sexual satisfaction

In bivariate analysis, sexual satisfaction was significantly lower in survivors who were male and who had received chemotherapy, radiation, or hormone therapy. Prostate and colon cancer survivors scored lowest in sexual satisfaction (Table 4).

**Table 4:** Sexual satisfaction in relation to demographic and cancer specific variables.

Variable	Sexual satisfaction sum score M(SD)	p	η^2^
Time since diagnosis		.170	0.002
5 years	2.01 (0.56)		
10 years	2.06 (0.55)		
Sex		< .001*	0.013
Male	1.97 (0.57)		
Female	2.09 (0.52)		
Age cohorts		.496	0.002
18–49	2.10 (0.50)		
50–70	2.03 (0.55)		
71–85	2.01 (0.57)		
Cancer diagnosis		< .001*	0.059
Prostate	1.85 (0.58)		
Breast	2.08 (0.55)		
Gynaecological	2.12 (0.55)		
Head and neck	2.17 (0.50)		
Hematological	2.06 (0.52)		
Skin	2.25 (0.44)		
Kidney	2.15 (0.50)		
Colon	1.88 (0.55)		
Other	2.13 (0.54)		
Surgery			
Yes	2.03 (0.55)	.451	0.001
No	1.98 (0.61)		
Chemotherapy		.002*	0.014
Yes	1.97 (0.55)		
No	2.10 (0.57)		
Radiation		.026*	0.006
Yes	2.01 (0.56)		
No	2.10 (0.55)		
Hormone therapy			
Yes	1.89	< .001*	0.034
No	2.11		

η^2^: < 0.06—small effect, 0.06–0.14—medium effect, > 0.14—large effect, *significant on an Bonferroni-adjusted level of p < 0.00625.

There were small significant negative correlations between sexual satisfaction and chronic comorbidity index (*r* = − 0.27, *p* < 0.001), fatigue (*r* = − 0.35, p < 0.001), nausea/vomiting (*r* = − 0.13, p < 0.001), pain (*r* = − 0.23, p < 0.001), anxiety (*r* = − 0.23, *p* < 0.001), and depression (*r* = − 0.24, *p* < 0.001). Significant small to medium positive correlations were found between sexual satisfaction and social support (*r* = 0.16, *p* < 0.001), satisfaction with relationship (*r* = 0.24, *p* < 0.001) and all quality of life function scales (*r* = 0.23 to *r* = 0.37, *p* < 0.001) and global quality of life (*r* = 0.33, *p* < 0.001).

### Factors predicting sexual satisfaction

We performed a hierarchical regression with sexual satisfaction as the dependent variable (Table 5). The following associated factors were identified as having a positive influence on sexual satisfaction: female gender, not receiving hormone therapy, low chronic comorbidity index, fewer symptoms of fatigue, and high emotional and social functioning. After entering the scales measuring inter- and intrapersonal resources, all scales measuring physical symptom burden and psychological symptom burden were no longer significant. The highest variance explanation was found between the second and the third model (Change in R^2^ = 0.17).

**Table 5:** Multivariate hierarchical logistic regression of sexual satisfaction with associated factors.

Dependent variable: sexual satisfaction	Model 1		Model 2		Model 3		Model 4		Model 5	
	Beta	p	Beta	p	Beta	p	Beta	p	Beta	p
Sex	0.04	.384	0.09	.064	0.16***	< .001	0.18***	< .001	0.16***	< .001
Chemotherapy			− 0.07	.143	− 0.02	.627	0.01	.776	0.05	.302
Radiation			0.01	.869	0.03	.567	0.01	.878	− 0.00	.984
Hormone therapy			− 0.19***	< .001	− 0.13**	.006	− 0.12**	.008	− 0.10*	.031
Chronic comorbidity index					− 0.12*	.025	− 0.13*	.019	− 0.10	.073
Fatigue					− 0.24***	.000	− 0.07	.347	− 0.06	.464
Nausea/ vomiting					− 0.04	.453	− 0.00	.938	0.01	.822
Pain					− 0.02	.706	− 0.01	.900	0.04	.561
Physical functioning					0.10	.113	0.10	.127	0.00	.986
Anxiety							0.18	.075	0.17	.074
Depression							− 0.21	.041	− 0.19	.060
Emotional Functioning							0.18*	.013	0.09	.229
Cognitive Functioning							0.08	.136	0.04	.499
Partnership satisfaction									0.07	.108
Social support									0.05	.198
Social Functioning									0.21***	< .001
Roles Functioning									0.05	.509
Global quality of life									0.11	.090
Adjusted R^2^	0.00		0.04		0.20		0.23		0.27	
Change in R^2^	0.00		0.04		0.17		0.03		0.05	
Change in F	0.759		6.873***		20.217***		4.910***		5.936***	
Sig. Change in F	.384		< .001		< .001		< .001		< .001	

R^2^ = Nagelkerke R^2^, p = significance, *p < .05, **p < .01, ***p < .001.

## Discussion

The purpose of the present study was to examine long-term cancer survivors' sexual satisfaction and the factors that are associated with it. We found that respondents experienced less sexual satisfaction compared to the time before the cancer diagnosis. A German survey with 4955 men and women on their health, sexual activity and sexual satisfaction (GeSiD) also found that respondents who describe their health as only fair or bad, who define themselves as chronically ill or disabled, or who suffer from one or more of a list of specific health problems are more likely to be dissatisfied with their sexual life than those who state that they are in better health^47^.

However, since respondents rated their sexual satisfaction retrospectively, we had no information about their actual baseline satisfaction. There is also ample evidence that sexual satisfaction generally decreases with age and relationship duration^48,49^. Therefore, the aforementioned decrease may overlap with the occurrence of sexual dissatisfaction as a late and long-term consequence of cancer. No differences in sexual satisfaction were noted between respondents living in a partnership or without a partner and between the two cohorts 5 and 10 years after diagnosis. The only difference was in satisfaction with physical attractiveness, with the 10-year cohort being more satisfied. Another study by Dorfman et al. also failed to observe an effect of partnership status on sexual satisfaction^50,^. Time since diagnosis also did not play a role in respondents' sexual satisfaction. Both cohorts already achieved survival of at least 5 years after diagnosis. The time span between 5 and 10 years may not play a critical role in determining changes in sexual satisfaction among survivors.

The results revealed further that sexual satisfaction was both associated with physical and psychological symptom burden. For psychological distress the bivariate analysis showed that the fewer symptoms of anxiety and depression survivors experienced, the more satisfied they were with their sex life. Akyol et al. found that sexual dissatisfaction was significantly higher in Turkish colorectal cancer patients with high anxiety scores and that symptom scale scores of the patients with high anxiety scores were significantly higher than that of patients with low anxiety scores^51^. Another study with 232 women with epithelial ovarian cancer found that depression was negatively correlated with sexual satisfaction and positively correlated with sexuality discomfort^52^. Physical symptom burden and sexual satisfaction were negatively correlated in our study. That is, the more limitations survivors experienced due to the comorbidities and the more physical symptoms of fatigue, nausea/vomiting and pain they had, the less satisfied they were with their sex life. This is in line with findings from a British national survey on a cancer-free population^53^ where sexual satisfaction and both the number and the type of comorbidities were negatively correlated. In a Chinese study, 3996 cancer survivors were asked about their sexual satisfaction, which was significantly associated with both the number and type of comorbidities^32^. Regarding our regression model, the highest variance explanation was found between the second and the third model. That is, physical symptom burden as represented by chronic comorbidity index, symptom scales (fatigue, pain, nausea/vomiting) and physical functioning had the greatest impact on sexual satisfaction.

For inter- and intrapersonal resources, the bivariate analyses of our study identified a positive correlation between factors such as social support, relationship satisfaction, and functioning levels including global quality of life, and the outcome of sexual satisfaction. Previous research among cancer survivors found that higher sexual satisfaction was significantly associated with higher relationship satisfaction^31^. Our correlation was relatively small and in regression analyses, we found no such effect. There is evidence that the correlation between sexual satisfaction and relationship satisfaction is weaker for those in long-term relationships^54^. We did not control whether participants were in a short or long-term relationship at the time of the survey. Relationship length could be a valuable factor to investigate in further studies.

We found that survivors did not address sexual issues, nor did they felt the need to discuss them. Given the many survivors who express dissatisfaction with sexuality, at least the opportunity to talk about it should be provided. We did not ask respondents whether their physicians had provided them with information on possible side effects of cancer treatment on their sexuality or other relevant concerns regarding survivors’ sexual health. Patients should receive educational materials with cancer-specific information on sexual problems, including guidance on topics such as resumption of sexual intercourse after abstinence, ways to increase sexual desire and alternative ways to feel sexual pleasure and material on how to communicate this topic (e.g. within the partnership)^55^.

### Study strengths and limitations

Our study is based on a large and representative cohort of cancer survivors, as access was through a cancer registry, which ensured validated diagnostic information and an excellent representation of cancer diagnosis in Germany. Another strength of our study is that the sample represents a wide age range (18–85 years) and a balanced gender ratio.

However, our study also has limitations. As we examined sexual satisfaction in a cross-sectional setting, this does not allow interferences on causality. Thus further studies on longitudinal effects should be conducted.

The frequency of occurrence estimation was based on self-reports. Sexual health and its vulnerability is an issue prone to stigmatization. It is also possible that self-reported data is biased towards underestimation or is subject to social acceptability bias. It should be noted, however, that this problem may be exacerbated in the context of face-to-face interviews with patients. As such, the assessment by self-report may provide comparatively more valid data.

Because the questionnaire used for our study was a newly developed instrument, normative data from the population were not available. Especially for the validation of this new sexuality questionnaire, it would therefore be interesting to present the questions to a cancer-free sample. This could also provide insights as to whether the associations found differ between cancer survivors and the general population.

### Clinical implications

Nearly half of all respondents were less satisfied with their sexuality compared to the time before cancer diagnosis. Thus, lower sexual satisfaction seems to be present even up to 10 years after cancer diagnosis. At the same time, sexuality is still a major taboo subject. This is most evident in a lack of communication about this specific topic in clinical practice. Sexual satisfaction was associated with physical and psychosocial symptom burden. Managing and reducing cancer survivors' comorbidities manifesting as physical and psychological symptom burden is important for improving their sexual satisfaction. Health care providers should understand, evaluate and treat sexual health issues, including those of long-term cancer survivors. Further research is needed to examine the factors that influence sexual satisfaction in cancer survivors in longitudinal studies.

## Supplementary Information

Supplementary Information 1.

Supplementary Information 2.

## Data Availability

The datasets generated during and/or analysed during the current study are available from the corresponding author on reasonable request.
