# Supplementary material for: Physical and psychosocial factors associated with sexual satisfaction in long-term cancer survivors 5 and 10 years after diagnosis
Source: Sci Rep. 2023 Feb 3;13:2011. doi: 10.1038/s41598-023-28496-1 (PMC9898518; doi:10.1038/s41598-023-28496-1)
Supplement: Supplementary file 1 — Supplementary Information 1. [file 41598_2023_28496_MOESM1_ESM.docx]

| **Information on other diseases** _CE_ | | | | | | |
| --- | --- | --- | --- | --- | --- | --- |
| You can find a list of common **chronic diseases** here. For each condition, please indicate whether you currently have that condition. If you have this condition, please rate how much it affects your daily activities.  1 means "not at all", 5 means "very much", in between you can grade the degree of your limitation. | | | | | | |
|  | I do not have this condition | I have this condition and it limits my daily activities ... | | | | |
|  |  | *not at all* |  | | | *very much* |
| 1. Hypertension | ☐_0_ | ☐_1_ | ☐_2_ | ☐_3_ | ☐_4_ | ☐_5_ |
| 1. Asthma | ☐_0_ | ☐_1_ | ☐_2_ | ☐_3_ | ☐_4_ | ☐_5_ |
| 1. Lung diseases (e.g. chronic bronchitis) | ☐_0_ | ☐_1_ | ☐_2_ | ☐_3_ | ☐_4_ | ☐_5_ |
| 1. Diabetes (diabetes) | ☐_0_ | ☐_1_ | ☐_2_ | ☐_3_ | ☐_4_ | ☐_5_ |
| 1. Thyroid disease | ☐_0_ | ☐_1_ | ☐_2_ | ☐_3_ | ☐_4_ | ☐_5_ |
| 1. Chronic back pain | ☐_0_ | ☐_1_ | ☐_2_ | ☐_3_ | ☐_4_ | ☐_5_ |
| 1. Rheumatism | ☐_0_ | ☐_1_ | ☐_2_ | ☐_3_ | ☐_4_ | ☐_5_ |
| 1. Joint wear and tear | ☐_0_ | ☐_1_ | ☐_2_ | ☐_3_ | ☐_4_ | ☐_5_ |
| 1. Osteoporosis | ☐_0_ | ☐_1_ | ☐_2_ | ☐_3_ | ☐_4_ | ☐_5_ |
| 1. Intestinal disorders (e.g. irritable bowel syndrome, ulcerative colitis, etc.) | ☐_0_ | ☐_1_ | ☐_2_ | ☐_3_ | ☐_4_ | ☐_5_ |
| 1. Stomach complaints (e.g. stomach ulcers, gastritis or heartburn) | ☐_0_ | ☐_1_ | ☐_2_ | ☐_3_ | ☐_4_ | ☐_5_ |
| 1. Kidney disease | ☐_0_ | ☐_1_ | ☐_2_ | ☐_3_ | ☐_4_ | ☐_5_ |
| 1. Sensory disturbances (tingling, numbness or burning in the legs or hands / polyneuropathy) | ☐_0_ | ☐_1_ | ☐_2_ | ☐_3_ | ☐_4_ | ☐_5_ |
| 1. Heart disease (e.g. heart attack, heart failure, bypass surgery) | ☐_0_ | ☐_1_ | ☐_2_ | ☐_3_ | ☐_4_ | ☐_5_ |
| 1. Stroke | ☐_0_ | ☐_1_ | ☐_2_ | ☐_3_ | ☐_4_ | ☐_5_ |
| 1. Neurological diseases (e.g. Parkinson's disease, dementia) | ☐_0_ | ☐_1_ | ☐_2_ | ☐_3_ | ☐_4_ | ☐_5_ |
| 1. Diseases of the eyes (e.g. cataract/ glaucoma) | ☐_0_ | ☐_1_ | ☐_2_ | ☐_3_ | ☐_4_ | ☐_5_ |
| 1. Mental illnesses (e.g. depression) | ☐_0_ | ☐_1_ | ☐_2_ | ☐_3_ | ☐_4_ | ☐_5_ |
| If yes, which ones:  ……………………………………………………………………. | | | | | | |
