# Supplementary material for: Physical and psychosocial factors associated with sexual satisfaction in long-term cancer survivors 5 and 10 years after diagnosis
Source: Sci Rep. 2023 Feb 3;13:2011. doi: 10.1038/s41598-023-28496-1 (PMC9898518; doi:10.1038/s41598-023-28496-1)
Supplement: Supplementary file 2 — Supplementary Information 2. [file 41598_2023_28496_MOESM2_ESM.docx]

**Questions about sexuality and partnership** (SEX)

| Many patients report changes in their sexuality and partnership. We know that this can be an intimate topic for many people, but at the same time an important one. The following questions therefore refer to various aspects of sexuality and partnership **at the present time.** |
| --- |

|  | | extremely dissatisfied | dissatisfied | neither/ nor | satisfied | extremely satisfied |
| --- | --- | --- | --- | --- | --- | --- |
|  | With my physical attractiveness I am ... | ☐_1_ | ☐_2_ | ☐_3_ | ☐_4_ | ☐_5_ |

##### **If you are currently living in a partnership:**

|  | | extremely dissatisfied | dissatisfied | neither/ nor | satisfied | extremely satisfied |
| --- | --- | --- | --- | --- | --- | --- |
|  | How satisfied are you with your sexual relationship with your partner overall? | ☐_1_ | ☐_2_ | ☐_3_ | ☐_4_ | ☐_5_ |
|  | | always | almost always | sometimes | rarely | never |
|  | How often do physical symptoms (e.g., pain) affect your ability to feel pleasure during sexual acts? | ☐_1_ | ☐_2_ | ☐_3_ | ☐_4_ | ☐_5_ |
|  | How often do emotional stresses (e.g., sadness) affect your ability to feel pleasure during sexual acts? | ☐_1_ | ☐_2_ | ☐_3_ | ☐_4_ | ☐_5_ |

##### **If you are currently living without a partner:**

|  | | do not agree at all | do not agree | neither/  nor | do agree | agree completely |
| --- | --- | --- | --- | --- | --- | --- |
|  | I am satisfied with my sex life as it is. | ☐_1_ | ☐_2_ | ☐_3_ | ☐_4_ | ☐_5_ |
|  | I am satisfied with my life without partnership as it is. | ☐_1_ | ☐_2_ | ☐_3_ | ☐_4_ | ☐_5_ |
|  | I wish for a new partnership. | ☐_1_ | ☐_2_ | ☐_3_ | ☐_4_ | ☐_5_ |

**Changes in sexuality compared to before you had cancer:**

|  | | much worse | rather worse | neither/  nor | rather better | much better |
| --- | --- | --- | --- | --- | --- | --- |
|  | the satisfaction with sexuality is now ... | ☐_1_ | ☐_2_ | ☐_3_ | ☐_4_ | ☐_5_ |
|  | the communication about sexuality is now ... | ☐_1_ | ☐_2_ | ☐_3_ | ☐_4_ | ☐_5_ |

**Desire for counseling on sexual problems**

|  | | yes | no |
| --- | --- | --- | --- |
|  | Have you discussed problems or concerns about your sexuality with a doctor or psychologist? | ☐_1_ | ☐_0_ |
|  | Do you have or have you felt the need to discuss problems or concerns about your sexuality with a doctor or psychologist? | ☐_1_ | ☐_0_ |
